# Dvl2-Dependent Activation of Daam1 and RhoA Regulates Wnt5a-Induced Breast Cancer Cell Migration

**DOI:** 10.1371/journal.pone.0037823

**Published:** 2012-05-24

**Authors:** Yichao Zhu, Yinhui Tian, Jun Du, Zhenzhen Hu, Ling Yang, Jiaojing Liu, Luo Gu

**Affiliations:** 1 State Key Laboratory of Reproductive Medicine, Nanjing Medical University, Nanjing, Jiangsu, China; 2 Cancer Center, Nanjing Medical University, Nanjing, Jiangsu, China; 3 Department of Physiology, Nanjing Medical University, Nanjing, Jiangsu, China; 4 Department of Cardiology, Third Affiliated Hospital of Suzhou University, Changzhou, Jiangsu, China; National Cancer Center, Japan

## Abstract

**Background:**

The Dishevelled (Dvl) and Dishevelled-associated activator of morphogenesis 1 (Daam1) pathway triggered by Wnt5a regulates cellular polarity during development and tissue homoeostasis. However, Wnt5a signaling in breast cancer progression remains poorly defined.

**Methodology/Principal Findings:**

We showed here that Wnt5a activated Dvl2, Daam1 and RhoA, and promoted migration of breast cancer cells, which was, however, abolished by Secreted Frizzled-related protein 2 (sFRP2) pretreatment. Dominant negative Dvl2 mutants or Dvl2 siRNA significantly decreased Wnt5a-induced Daam1/RhoA activation and cell migration. Ectopic expression of *N*-Daam1, a dominant negative mutant, or Daam1 siRNA remarkably inhibited Wnt5a-induced RhoA activation, stress fiber formation and cell migration. Ectopic expression of dominant negative RhoA (N19) or C3 exoenzyme transferase, a Rho inhibitor, decreased Wnt5a-induced stress fiber formation and cell migration.

**Conclusions/Significance:**

Taken together, we demonstrated for the first time that Wnt5a promotes breast cancer cell migration *via* Dvl2/Daam1/RhoA.

## Introduction

Despite advances in the early diagnosis and adjuvant treatment of breast carcinoma, this disease still remains the most common malignancy of women worldwide [1], [2]. Several improvements in understanding the molecular pathology of metastatic breast cancer have been achieved over the past decade. However, the molecular mechanisms underlying this malignancy are still largely unknown. For this reason, elucidating the signaling pathways involved in the metastatic cascade is a key goal for developing novel effective therapeutics aimed at reducing cancer mortality rates.

The Wnt signaling has historically been divided into two classes: the canonical (β-catenin dependent) and non-canonical (β-catenin independent) signaling pathway. The canonical Wnt signaling pathway has been implicated in promoting malignant transformation and tumor progression [3], [4]. Wnt/TCF signaling program, for example, has been reported to be capable of enhancing the competence of lung adenocarcinoma cells to colonize the bones and the brain [5], while very limited studies have been done on the role and mechanisms of non-canonical Wnt signaling in tumor progression. Wnt5a is a representative of Wnt proteins that activates non-canonical Wnt signaling. The Wnt/planar cell polarity (PCP) pathway triggered by Wnt5a activates small Rho-GTPases and regulates the cytoskeletal architecture and cellular polarity during development and tissue homoeostasis [6], [7], [8]. Wnt5a is classified as a non-transforming Wnt family member that plays complicated roles in oncogenesis and cancer metastasis. In malignant melanoma and gastric cancer, Wnt5a actually promotes cancer progression [9], [10], [11]. Conversely, Wnt5a functions as a tumor suppressor in colon, thyroid, and hepatocellular cancers [12], [13], [14]. In the breast, dysregulated Wnt signaling appears to occur by autocrine mechanisms [15], [16], [17]. Interference with autocrine Wnt signaling can block *in vitro* proliferation of many human breast cancer cell lines [16], [17]. The non-transforming Wnt5a can inhibit breast epithelial cell migration [18] and predicts longer disease-free survival for patients with breast cancer [19]. In contrast to the hypothesis that Wnt5a functions as a tumor suppressor, Wnt5a secreted by macrophages is proved to be essential for macrophage-induced invasiveness of breast cancer cells [20].

These promiscuous viewpoints of Wnt5a in breast cancer progression led us to further elucidate the function of Wnt5a, and investigate the underlying mechanisms whereby cell migration is regulated. Here, we demonstrated for the first time that Wnt5a promotes the migration of breast cancer cells, and we report on the mechanisms whereby Wnt5a/PCP signaling regulates cell migration. Wnt5a signaling directly activates RhoA, which requires Dishevelled 2 (Dvl2) and Dishevelled-associated activator of morphogenesis 1 (Daam1).

## Materials and Methods

### Plasmids and transient transfections

The plasmids pCB6-GFP-RhoA-WT, V14 and N19 were kindly provided by Dr. Stéphane ORY (Institute of Cellular and Integrative Neurosciences, University of Strasbourg, France). Dr. Marc Fiedler (MRC Laboratory of Molecular Biology, Cambridge, UK) generously provided the construct of human full-length Dvl2. The human full-length Daam1 was kindly gifted from Dr. Raymond Habas (Departments of Biochemistry and Pharmacology and Cancer Institute of New Jersey, USA). Mutant fragments of Dvl2 and Daam1 were generated by restriction digestion or a PCR approach and subcloned in pEGFP-N1 or pCS2 vectors. Details of plasmids are available upon request.

MDA-MB-231 and MCF-7 cell lines (ATCC, Manassas, VA) were grown in Dulbecco's modified Eagle's medium (DMEM, high glucose) (Hyclone, Thermo Scientific, Waltham, MA) supplemented with 10% (v/v) fetal bovine serum (FBS) (Hyclone) in a humidified incubator at 37°C with 5% CO_2_. The cells were seeded in 6-well plates (Costar, Corning, NY) and cultured to 80∼90% confluence, and then transiently transfected with plasmids using Lipofectamine 2000 Reagent (Invitrogen, Carlsbad, CA) in serum-free OPTI-MEM according to the manufacturer's instructions. The cells were switched to fresh medium containing 10% FBS 6 h after the transfection and cultured for 48 h. The cells transfected with Dvl2, Daam1 and RhoA constructs were used for analyzing the expression of these proteins and cell migration.

### Wound-healing assay

MDA-MB-231 cells were plated onto 96-well cell culture clusters (Costar) and grown to confluence, and then serum-starved for 24 h. Recombinant sFRP2 (R&D Systems, Minneapolis, MN) and C3 exoenzyme (Enzo Life Sciences, Plymouth Meeting, PA) were used 60 min before the scratch was made. The cells transfected with indicated plasmids were switched to fresh medium containing 10% FBS 6 h after the transfection and cultured for 48 h, and then serum-starved for 24 h. The monolayer cells were scratched manually with a plastic pipette tip, and after two washes with PBS, the wounded cellular monolayer was allowed to heal for 4 h in DMEM containing 500 ng/mL recombinant Wnt5a (rWnt5a) (R&D Systems). Photographs of central wound edges per condition were taken at time 0 and at the indicated time points using PowerShot G10 camera (Canon, Tokyo, Japan).

### Cell migration assays

Cell migration was assessed in a modified Boyden chamber (Costar), in which the two chambers were separated by a polycarbonate membrane (pore diameter, 8.0 µm). Boyden chamber wells were coated with human collagen I (20 µg/mL) for 1 h at 37°C. MDA-MB-231 or MCF-7 cells were grown to subconfluence in tissue culture plates and then detached, after which they were centrifuged and rendered into single cell suspensions in serum-free culture medium supplemented with 5 µg/mL BSA. The suspensions containing 5×10^4^ cells were added to wells with a membrane placed in the bottom. For MDA-MB-231 cell migration, medium containing 500 ng/mL rWnt5a was added to the upper and lower compartment of the Boyden chamber. For MCF-7 cell migration, serum-free medium was added to the upper and lower compartment of the Boyden chamber. The cells were allowed to migrate for the indicated periods of time at 37°C in this assay. Thereafter, the medium was discarded, stationary cells were removed with a cotton-tipped applicator and the membranes were cut out of the chamber and stained with 0.5% crystal violet. The response was evaluated in a light microscope by counting the number of cells that had migrated into the membrane.

### RNAi

For gene knockdown, small interfering RNA (siRNA) duplexes specific for Dvl2 (On-Target Plus: 5′-GUGAGAGCUACCUAGUCAATT-3′ and 5′-CGCUAAACAUGGAGAAGUATT-3′; GenePharma, Shanghai, China; GenBank/EMBL/DDBJ accession No. NM_004422), and Daam1 (On-Target Plus: 5′-GCUGUAUAAAGGCGUUAAUTT-3′ and 5′-GAGCUCAGAAUUGCAACAUTT-3′; GenePharma; GenBank/EMBL/DDBJ accession No. NM_014992) were transfected into MDA-MB-231 cells using Lipofectamine 2000 Reagent as described in the previous section. Knockdown efficiency was evaluated 48 h after transfection by measuring mRNA and protein levels in cell lysates using RT-PCR or immunoblotting.

### Immunoblotting analysis

Subconfluent cells were washed twice with PBS, and then lysed with ice-cold RIPA lysis buffer (50 mmol/L Tris, 150 mmol/L NaCl, 1% Triton X-100, 1% sodium deoxycholate, 0.1% SDS, 1 mmol/L sodium orthovanadate, 1 mmol/L sodium fluoride, 1 mmol/L EDTA, 1 mmol/L PMSF, and 1% cocktail of protease inhibitors) (pH7.4). The lysates were then clarified by centrifugation at 12,000 g for 20 min at 4°C. The whole cell and nucleonic fractions were prepared using Nuclear and Cytoplasmic Protein Extraction Kit (Beyotime, Nantong, China). The protein extracts were separated by 8, 10, or 12% SDS-PAGE. The immunoblotting procedure was performed as described [21] and the following antibodies were used: anti-GAPDH, β-actin (Sigma, St. Louis, MO), Dvl2, Daam1 (Santa Cruz Biotechnology, Santa Cruz, CA), Wnt5a (Cell Signaling Technology, Danvers, MA), RhoA (Abcam, Cambridge, MA) and β-catenin (Bioworld Technology, St. Louis Park, MN) antibodies. Protein bands were detected by incubation with horseradish peroxidase-conjugated antibodies and visualized with ECL reagent (Thermo Scientific, Rockford, IL).

### Determination of Dvl2 phosphorylation status

Equal volumes of total cellular protein of MDA-MB-231 cells were treated with phosphatase (Beyotime), phosphatase and phosphatase inhibitor (50 mmol/L EDTA) at 37°C for 1 h. Then, these samples were analyzed by blotting with anti-Dvl2 antibody. Total cellular proteins were incubated with anti-Dvl2 and protein A/G-agarose beads (Pierce, Rockfrod, IL) at 4°C for 24 h, and then were analyzed by blotting with anti-phosphotyrosine, anti-phosphoserine, anti-phosphothreonine antibodies (Millipore).

### Pulldown assays

For detection of active RhoA, equal volumes of total cellular protein were incubated with GST-RBD (gifted from Dr. Keith Burridge, Department of Cell and Developmental Biology, University of North Carolina, Chapel Hill, NC) beads captured on MagneGST Glutathione Particles (Promega, Madison, WI) at 4°C with constant rotation for 90 min. The beads were washed three times with washing buffer (4.2 mmol/L Na_2_HPO_4_, 2 mmol/L KH_2_PO_4_, 280 mmol/L NaCl, and 10 mmol/L KCl, pH7.2). At the end of this period, beads were captured by the magnet in a magnetic stand. After washing three times with ice-cold buffer, beads were resuspended in Laemmli buffer, boiled, and subjected to immunoblotting analysis. SDS-PAGE and immunoblotting were performed using standard methods. For detection of active Daam1, GST-RhoA beads were incubated with 0.1 mmol/L GTPγS (Sigma) at 30°C for 15 min with constant agitation. The other procedures were described as above.

### Reverse transcription PCR (RT-PCR)

Total RNAs were isolated with TRIzol reagent (Invitrogen). First-strand cDNAs were synthesized using total RNAs, avian myeloblastosis virus (AMV) reverse transcriptase (Promega), and an oligo(dT) primer. Primers used for PCR amplification were as follows: GAPDH: 5′-TGAACGGGAAGCTCACTGG-3′ (sense) and 5′-TCCACCACCCTGTTGCTGTA-3′ (antisense); Wnt5a: 5′-CTTCGCCCAGGTTGTAATTGAAGC-3′ (sense) and 5′-CTGCCAAAAACAGAGGTGTTATCC-3′ (antisense); Dvl2: 5′-CATCCTTCCACCCTAATGTGTCC-3′ (sense) and 5′-GTCCCCCAGGCTGGTACTCT-3′ (antisense); Daam1: 5′-AAATTGAAACGGAATCGCAAAC-3′ (sense) and 5′-GCAAGGCAGTGTAATGAAACG-3′ (antisense). RT-PCR was done for 26 cycles with each cycle for 30 sec at 94°C, 40 sec at 58°C, and 40 sec at 72°C. The PCR products were resolved by electrophoresis on 1% agarose gel. Images of electrophoresis were taken using the ChemiDOC XRS Imaging system (BIO-RAD Laboratories, Hercules, CA).

### Actin cytoskeleton staining and immunofluorescence

Transfected cells were fixed in 4% paraformaldehyde in PBS for 20 min, permeabilized in 0.2% Triton X-100 and blocked in PBS containing 1% BSA for 1 h at room temperature. F-actin was stained with FITC-labeled phalloidin (5 µg/mL) (Sigma) for 40 min at room temperature. After wash with PBS, the cover slips were mounted on glass slides with DAPI Fluoromount G (Southern Biotech, Birmingham, AL). The images were acquired with a fluorescence microscope (Olympus, Tokyo, Japan). For semi-quantification of actin fibers, a baseline of ten actin fibers/cell was used. Cells containing more or fewer than ten fibers were scored as an increase or decrease, respectively.

### MTT assays

The 3-(4,5-dimethylthiazol-2-yl)-2,5-diphenyltetrazolium bromide (MTT) assay was used for the assessment of cell proliferation. MDA-MB-231 cells were seeded on 96-well plates in 100 µL medium for each well, cultured at 37°C for 24 h, and then were made quiescent by serum starvation for 24 h. Then, the cells were cultured for 24 to 48 h. Before each time point, 20 µL MTT solution was added to each well followed by incubation at 37°C for 4 h. After removal of the medium, 150 µL dimethylsulfoxide (DMSO) was added to each well. After gentle shaking, absorbance at 490 nm was measured.

### Statistical analysis

The data were analyzed using Student's t-test by SPSS statistical software package. All the results were expressed as mean ± s.d. For all analyses a two-sided *P* value of less than 0.05 was deemed statistically significant.

## Results

### Wnt5a stimulates breast cancer cell migration *in vitro*

**Figure 1:**
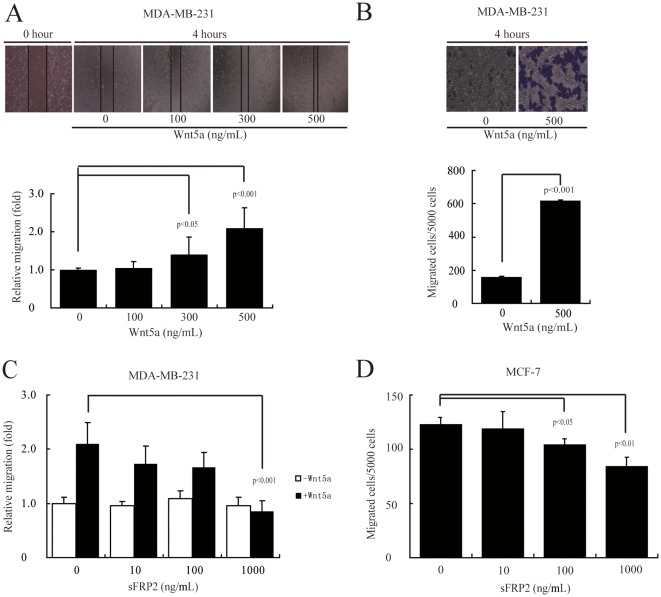
Wnt5a promotes MDA-MB-231 and MCF-7 cell migration. (A, B) MDA-MB-231 cells were stimulated by rWnt5a at the indicated doses for 4 h. The cell motility rate was measured by wound healing assays (A) or Boyden chamber assays (B). Magnification, ×100 (A) and ×200 (B). (C) MDA-MB-231 cells were preincubated with Wnt5a inhibitor (sFRP2) for 1 h at the indicated doses, and migration in response to rWnt5a (500 ng/mL, for 4 h) was measured by wound healing assays. (D) MCF-7 cells were preincubated with sFRP2 for 1 h at the indicated doses, and migration was measured by Boyden chamber assays after 48 h. Results are presented as mean ± s.d. of 5 independent experiments in (A) to (D).

To assess the effect of Wnt5a on breast cancer cell migration, we treated MDA-MB-231 cells with different doses of recombinant Wnt5a (rWnt5a), and measured the migration rate by wound healing assay. MDA-MB-231 cells expressed lower levels of Wnt5a than breast cancer cell line MCF-7 (Fig. S1), which is consistent with the previous report [22]. The rWnt5a activity was analyzed by immunoblotting to exclude that this recombinant protein has not been deglycosylated (Fig. S2). We found that Wnt5a had a potent stimulatory effect on MDA-MB-231 cell migration (Fig. 1A). An approximately 2-fold increase in cell migration was observed in cells treated with 500 ng/mL rWnt5a (Fig. 1A and Fig. S3). By using Boyden chamber assay, we found that more MDA-MB-231 cells incubated with rWnt5a migrated through the membrane than the untreated cells (Fig. 1B). We next determined whether Wnt5a promoted the proliferation of MDA-MB-231 cells by MTT assays. Treatment of MDA-MB-231 cells with 500 ng/mL rWnt5a resulted in an insignificant promotion of cell growth (Fig. S4). Accordingly, 500 ng/mL rWnt5a was used for further studies to identify the mechanism whereby changes in the migration of MDA-MB-231 cells were induced.

Pre-incubation of Secreted Frizzled-related protein 2 (sFRP2), an antagonist that directly binds to Wnts [23], abolished rWnt5a (500 ng/mL)-stimulated MDA-MB-231 cell migration (Fig. 1C). SFRP2 does not alter the nuclear translocation of β-catenin in MDA-MB-231 cells (Fig. S5). Additionally, low dose of sFRP2 abolished MCF-7 cell migration (Fig. 1D). Taken together, these experiments demonstrated that Wnt5a stimulated MDA-MB-231 and MCF-7 cell migration *in vitro*.

### Dvl2 activation is required for Wnt5a-induced cell migration

**Figure 2:**
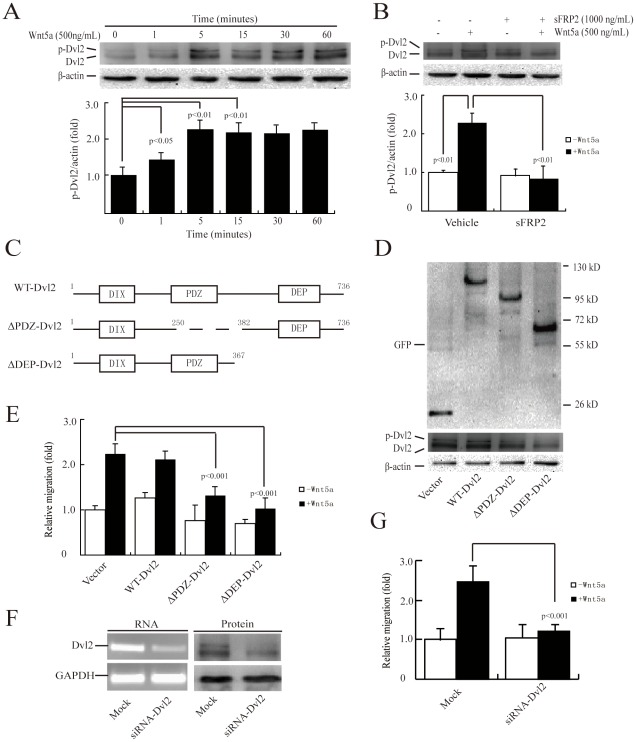
Dvl2 activation is required for Wnt5a-induced MDA-MB-231 cell migration. (A, B) Dvl2 activation was induced by Wnt5a (A) and blocked by sFRP2 (B). Serum-starved MDA-MB-231 cell monolayers were incubated with 500 ng/mL rWnt5a for 0–60 min (A), or treated with 1000 ng/mL sFRP2 for 1 h prior to 500 ng/mL rWnt5a treatment for 5 min (B). Cell lysates were assayed for phosphorylated Dvl2 by immunoblotting analyses with anti-Dvl2 and β-actin antibodies. Results are presented as mean ± s.d. of 3 independent experiments in (A) and (B). (C) The domain structures of Dvl2 and two mutants. Schematic representations of wild-type Dvl2, ΔPDZ-Dvl2 (lacking the PDZ domain), and ΔDEP-Dvl2 (lacking the DEP domain). Residue numbers above domains denote the domain boundaries. (D) Expression of empty vector, WT-Dvl2, ΔPDZ-Dvl2 or ΔDEP-Dvl2 was verified using total protein from cells and immunoblotted using anti-GFP antibody. (E) Overexpression of ΔPDZ-Dvl2 and ΔDEP-Dvl2 in cells abolished Wnt5a-induced cell migration. MDA-MB-231 cells transiently transfected with EGFP-tagged empty vector, WT-Dvl2, ΔPDZ-Dvl2 or ΔDEP-Dvl2 were incubated in the absence or presence of 500 ng/mL rWnt5a. The cell migration rate was determined by wound healing assays. (F) Efficiency of gene knockdown was analyzed by RT-PCR (left) and immunoblotting (right) for Dvl2. MDA-MB-231 cells were transfected with control (Mock) or Dvl2 siRNAs. Total mRNA or protein extracts from MDA-MB-231 transfected with control (Mock) or Dvl2 siRNA were analyzed by RT-PCR and immunoblotting for Dvl2. The same assay was performed with GAPDH as a loading standard. (G) Dvl2 siRNA significantly inhibited cell migration. MDA-MB-231 cells transfected with control (Mock) or Dvl2 siRNA were subjected to a wound healing assay in the absence or presence of 500 ng/mL rWnt5a. Results are presented as mean ± s.d. of 5 independent experiments in (E) and (G).

In view of the fact that several Wnt proteins, such as Wnt5a, trigger Dvl2 phosphorylation in other cell types [24], [25], we speculated that Wnt5a may also promote Dvl2 phosphorylation in MDA-MB-231 cells. To confirm that the upper band corresponded to a more highly phosphorylated form of Dvl2, extracts of MDA-MB-231 cells were treated with phosphatase in the presence or absence of phosphatase inhibitors (Fig. S6). As expected, the shifted band of Dvl2 was the phosphorylated form (Fig. S6). Afterwards, Dvl2 showed visible signs of basal phosphorylation and elevated phosphorylation at 1 min after treatment with 500 ng/mL rWnt5a (Fig. 2A). Maximal phosphorylation was detected at 5 min after treatment (Fig. 2A). Pre-treatment with 1000 ng/mL sFRP2 blocked Wnt5a-induced Dvl2 phosphorylation (Fig. 2B), indicating that the effects observed were specifically induced by Wnt5a.

We next sought to determine whether Dvl2 activation was required for Wnt5a-mediated MDA-MB-231 cell migration. Dvl2 has three major functional domains: the DIX domain, the central PDZ domain, and the C-terminal DEP domain (Fig. 2C). The DIX domain is essential for β-catenin signaling but dispensable for PCP, whereas both the PDZ and DEP domains are required for PCP function [26], [27], [28], [29], [30]. We created a panel of Dvl2 mutants (Fig. 2C) and tested their roles in cell migration (Fig. 2E). The puncta of wild-type (WT) Dvl2 or mutants tagged with enhanced green fluorescent protein (EGFP) were transiently transfected into MDA-MB-231 cells (Fig. 2D). The expression efficiency of EGFP-tagged puncta was 60∼70% as observed under a fluorescence microscope. ΔPDZ-Dvl2 (lacking the PDZ domain) and ΔDEP-Dvl2 (lacking the DEP domain) were capable of retarding Wnt5a-induced cell migration, and the overexpression of WT-Dvl2 was not able to accelerate cell migration (Fig. 2E). These findings, thus, suggested a close correlation between two specific domains of Dvl2 and MDA-MB-231 cell migration.

We also used siRNA to knock down Dvl2 expression in breast cancer cells and checked whether Wnt5a-induced cell migration could be inhibited. The siRNA against human Dvl2 knocked down Dvl2 expression by more than 50% as assessed by RT-PCR and immunoblotting in MDA-MB-231 cells (Fig. 2F), which resulted in a significant reduction of Wnt5a-induced cell migration (Fig. 2G). Taken together, these experiments demonstrated that Wnt5a induced the migration of MDA-MB-231 cells by integrating the whole functional domains of Dvl2.

### Daam1 acts as a downstream target of Dvl2 and mediates Wnt5a-induced cell migration

**Figure 3:**
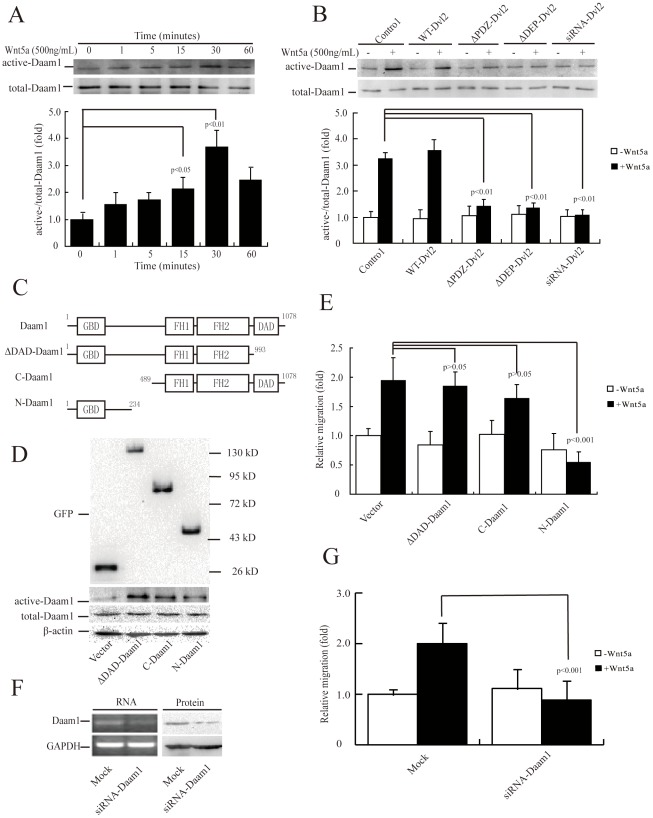
Daam1 is a downstream target of Dvl2 and its activation is required for Wnt5a-induced MDA-MB-231 cell migration. (A, B) Daam1 activation was induced by Wnt5a (A) and blocked by Dvl2 mutants or siRNA (B). Serum-starved MDA-MB-231 cell monolayers were incubated with 500 ng/mL rWnt5a for 0–60 min (A), or transiently transfected with Dvl2 mutants or siRNA, and then incubated with 500 ng/mL rWnt5a for 30 min (B). Cellular lysates were assayed for the active Daam1 by pulldown assay using a GST-RhoA as a bait. Results are presented as mean ± s.d. of 3 independent experiments in (A) and (B). (C) The domain structures of Daam1 and three mutants. Schematic representations of wild-type Daam1, ΔDAD-Daam1 (lacking the DAD domain), *C*-Daam1 (C-terminal of Daam1) and *N*-Daam1 (N-terminal of Daam1). Residue numbers above domains denote the domain boundaries. (D) Expression of empty vector, ΔDAD-Daam1, *C*-Daam1, or *N*-Daam1 was verified using total protein from cells and immunoblotted using anti-GFP antibody. (E) Overexpression of ΔDAD-Daam1 and *C*-Daam1 in cells maintained whereas *N*-Daam1 abolished Wnt5a-induced cell migration. MDA-MB-231 cells were transiently transfected with empty vector, ΔDAD-Daam1, *C*-Daam1 or *N*-Daam1. Cell migration rate was determined by wound healing assays, which were allowed to heal for 4 h, in the absence or presence of 500 ng/mL rWnt5a. (F) Efficiency of gene knockdown was analyzed by RT-PCR (left) and immunoblotting (right) for Daam1. MDA-MB-231 cells were transfected with Daam1 siRNAs or control (Mock). Total mRNA or protein extracts from MDA-MB-231 transfected with indicated materials were analyzed by RT-PCR and immunoblotting for Daam1. The same assay was performed with GAPDH as a loading standard. (G) Daam1 siRNA significantly inhibited cell migration. MDA-MB-231 cells transfected with indicated materials were subjected to a wound healing assay in the absence or presence of 500 ng/mL rWnt5a. Results are presented as mean ± s.d. of 5 independent experiments in (E) and (G).

Binding of Daam1 to Dvl and the small GTPase Rho has been shown to coordinate Wnt signaling cues in *Xenopus* [31]. We examined whether Daam1 was also activated by Wnt5a in MDA-MB-231 cells. Immunoblotting showed a visible sign of basal active Daam1 and a clear maximal effect after 30 min of rWnt5a treatment (Fig. 3A). We next examined whether Daam1 was the downstream target of Dvl2 in MDA-MB-231 cells. These Dvl2 mutants (ΔPDZ-Dvl2 and ΔDEP-Dvl2) and siRNA blocked Wnt5a-induced Daam1 activation (Fig. 3B), indicating that Daam1 was a downstream target of Wnt5a/Dvl2 signaling in MDA-MB-231 cells.

Because the C-terminal (*C*-Daam1), ΔDAD-Daam1 (lacking the DAD domain) and N-terminal (*N*-Daam1) domains of Daam1 exert constitutively active and dominant negative functions on PCP signaling, respectively (Fig. 3C) [31], [32], we compared the effects of these three mutants on cell migration. The puncta of Daam1 mutants tagged with EGFP were transiently transfected into MDA-MB-231 cells (Fig. 3D). The transfection efficiency of EGFP-tagged puncta were 60∼70% as observed under a fluorescence microscope. Abundant ΔDAD-Daam1 or *C*-Daam1 did not promote cell migration, while *N*-Daam1 was fully capable of retarding the migration of MDA-MB-231 cells (Fig. 3E). Thus, there was a close correlation between active Daam1 and MDA-MB-231 cell migration.

To analyze the role of endogenous Daam1 activation on Wnt5a-induced cell migration, we knocked down Daam1 expression using siRNA. The siRNA against human Daam1 reduced the mRNA and protein levels of Daam1 by more than 50%, as assessed by RT-PCR and immunoblotting (Fig. 3F) and significantly reduced Wnt5a-induced migration of MDA-MB-231 cells (Fig. 3G). Taken together, these experiments demonstrated that active Daam1 was the downstream target of Wnt5a/Dvl2 and its activation was required for Wnt5a-induced MDA-MB-231 cell migration.

### Wnt5a induces cell migration *via* RhoA activation

**Figure 4:**
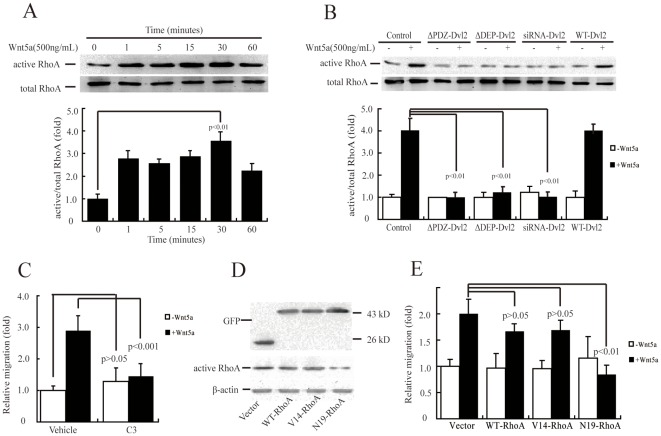
RhoA activation is essential for Wnt5a-induced MDA-MB-231 cell migration. (A, B) RhoA activation was induced by Wnt5a (A) and blocked by Dvl2 mutants or siRNA (B). Serum-starved MDA-MB-231 cell monolayers were incubated with 500 ng/mL rWnt5a for 0–60 min (A), or transiently transfected with Dvl2 mutants or siRNA, and then incubated with 500 ng/mL rWnt5a for 30 min (B). Cell lysates were assayed for active RhoA by pulldown assays. Results are presented as mean ± s.d. of 3 independent experiments in (A) and (B). (D) Expression of empty vector, WT-RhoA, V14-RhoA or N19-RhoA was verified using total protein from cells and immunoblotted using anti-GFP antibody. (C, E) Wnt5a-induced cell migration was abolished by C3 exoenzyme transferase (C) or N19-RhoA, a dominant negative mutant of RhoA (E). MDA-MB-231 cells were preincubated with Rho inhibitor C3 (10 ng/µL) for 1 h (C), or transiently transfected with empty vector, WT-RhoA, V14-RhoA, or N19-RhoA (E), and then incubated with 500 ng/mL rWnt5a for 4 h. Cell migration rate was determined by wound healing assay. Results are presented as mean ± s.d. of 5 independent experiments in (C) and (E).

We first investigated whether RhoA activation was induced by Wnt5a in MDA-MB-231 cells. Immunoblotting showed a visible sign of basal active RhoA and a maximal effect at 30 min after rWnt5a treatment (Fig. 4A). We next examined whether RhoA was downstream of Dvl2 in human breast cancer cells. Blocking Dvl2 with dominant negative mutants (ΔPDZ-Dvl2 and ΔDEP-Dvl2) or siRNA abolished Wnt5a-induced RhoA activation (Fig. 4B). These results indicated that RhoA functioned directly downstream of Dvl2 in MDA-MB-231 cells.

To study the role of RhoA activation in cell migration, we used C3 exoenzyme transferase, a Rho inhibitor (Fig. 4C, Fig. S7). Pre-incubation with 10 ng/µL C3 transferase for 1 h completely inhibited rWnt5a (500 ng/mL)-induced MDA-MB-231 cell migration (Fig. 4C). To further investigate the role of RhoA in cell migration, we transiently transfected MDA-MB-231 cells with GFP-tagged WT-RhoA, V14-RhoA (constitutively active mutant), or N19-RhoA (dominant negative mutant) (Fig. 4D). Overexpression of abundant WT-RhoA or V14-RhoA had no effect on cell migration, but overexpression of N19-RhoA completely abolished Wnt5a-induced cell migration (Fig. 4E). Thus, we concluded that RhoA activation was involved in Wnt5a-induced MDA-MB-231 cell migration.

### Dvl2-dependent RhoA activation requires Daam1 activity

**Figure 5:**
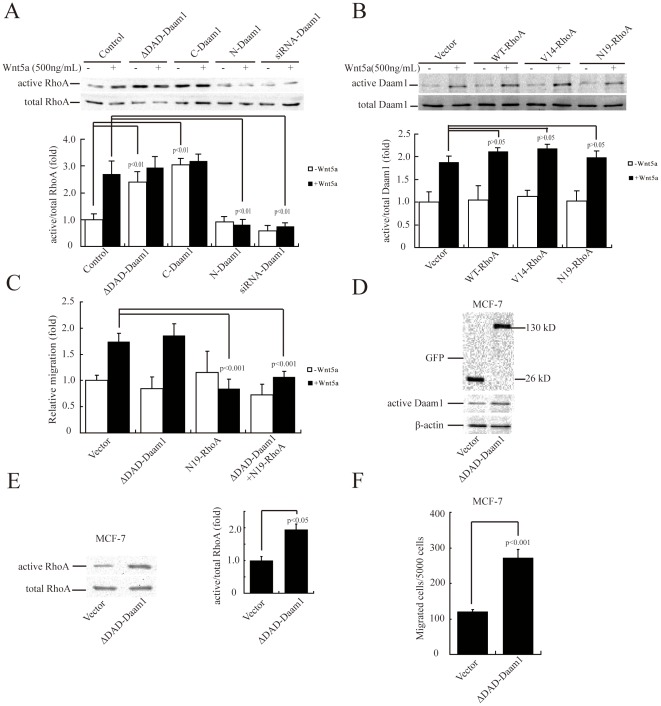
Daam1 activation is required for Wnt5a-induced RhoA activation. (A) ΔDAD-Daam1 and *C*-Daam1 induced while dominant negative mutant and Daam1 siRNA blocked RhoA activation. MDA-MB-231 cells were transiently transfected with Daam1 mutants or siRNAs, and then treated with 500 ng/mL rWnt5a for 30 min. Cells were lysed and quantitated for protein and equal amounts of lysates were assayed for active RhoA by pulldown assays. (B) RhoA did not change the activation of Daam1 with or without Wnt5a treatment. MDA-MB-231 cells were transiently transfected with WT-RhoA, V14-RhoA and N19-RhoA, and then treated with 500 ng/mL rWnt5a for 30 min. Equal amounts of lysates were assayed for active Daam1 by pulldown assays. (C) Overexpression of N19-RhoA abolished the Daam1-dependent cell migration. MDA-MB-231 cells were transiently co-transfected with N19-RhoA and ΔDAD-Daam1. Cells were subjected to a wound healing assay in the absence or presence of 500 ng/mL rWnt5a. (D) Expression of empty vector or ΔDAD-Daam1 was verified using total protein from MCF-7 cells and immunoblotted using anti-GFP antibody. (E) ΔDAD-Daam1 induced RhoA activation in MCF-7 cells. MCF-7 cells were transiently transfected with ΔDAD-Daam1. Cells were lysed and quantitated for protein and equal amounts of lysates were assayed for active RhoA by pulldown assays. Results are presented as mean ± s.d. of 3 independent experiments in (A), (B) and (E). (F) MCF-7 cells were stimulated by ΔDAD-Daam1. MCF-7 cells were transiently transfected with ΔDAD-Daam1 or empty vector, and migration was quantified by Boyden chamber assays after 48 h. Values are mean ± s.d. of 5 independent observations in (C) and (F).

Formin proteins, such as Daam1, regulate the actin dynamics by assembling actin filaments, which is also under the control of the Rho family GTPases [33], [34]. We found that ectopic expression of ΔDAD-Daam1 and *C*-Daam1 could induce RhoA activation (Fig. 5A), and overexpression of *N*-Daam1 or knockdown of Daam1 expression by siRNA blocked Wnt5a-induced RhoA activation (Fig. 5A), indicating that Daam1 acted as upstream of RhoA signaling in MDA-MB-231 cells.

To examine whether RhoA regulated Daam1 in MDA-MB-231 cells, we detected the activity of Daam1 after transiently transfecting MDA-MB-231 cells with WT-RhoA and RhoA mutants, respectively (Fig. 5B). There was no change in Daam1 activity after transfection in the absence or presence of Wnt5a. We further studied the coordinating role of Daam1 and RhoA activation in cell migration by transiently co-transfecting N19-RhoA and ΔDAD-Daam1 into MDA-MB-231 cells. We found that N19-RhoA and ΔDAD-Daam1 abolished Wnt5a-induced MDA-MB-231 migration (Fig. 5C).

Then, we examined whether Daam1 could activate RhoA in other human breast cancer cells. We transiently transfected MCF-7 cells, which express high levels of Wnt5a (Fig. S1), with ΔDAD-Daam1 (Fig. 5D) and found that ΔDAD-Daam1 enhanced RhoA activation (Fig. 5E). Based on the similar pattern of Daam1 and RhoA activation between MDA-MB-231 and MCF-7 cell lines, we speculated that active Daam1 can induce the migration of MCF-7. Indeed, overexpression of ΔDAD-Daam1 can promote MCF-7 migration (Fig. 5F). Together, these data strongly suggested that Daam1 was required to induce RhoA activation and participated in Wnt5a-induced MDA-MB-231 and MCF-7 migration.

**Figure 6:**
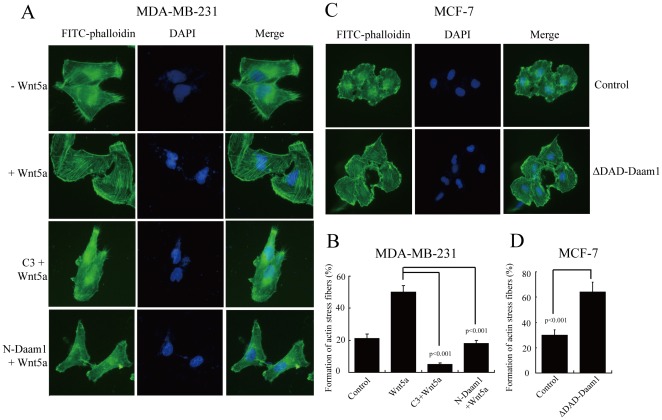
Daam1 participates in the rearrangement of stress fibers in MDA-MB-231 and MCF-7 cells. (A) *N*-Daam1 and C3 transferase disrupted the formation of actin stress fibers in MDA-MB-231 cells. MDA-MB-231 cells were transiently transfected with *N*-Daam1 or pre-treated with 10 ng/µL C3 transferase for 1 h. MDA-MB-231 cells as well as transiently transfected Daam1 mutants were grown on cover slips, and then incubated in culture medium containing 500 ng/mL rWnt5a for 4 h. Subsequently, cells were fixed and F-actin organization was analyzed by phalloidin staining. (C) ΔDAD-Daam1 enhanced the formation/maintenance of actin stress fibers in MCF-7. MCF-7 cells were transiently transfected with ΔDAD-Daam1, and then incubated in culture medium in the absence of Wnt5a. Magnification, ×400. (B, D) The percentage of formation of actin stress fibers was determined in MDA-MB-231 cells (B) and MCF-7 cells (D), as described in Materials and Methods. The average is shown as mean ± s.d.

Rho GTPases are key regulators of the actin cytoskeleton [35]. We performed fluorescent phalloidin staining to investigate the distribution pattern of F-actin in MDA-MB-231 and MCF-7 cells. We found that *N*-Daam1 disrupted the formation of actin stress fibers in MDA-MB-231 cells, similar to those with C3 transferase treatment (Fig. 6A and 6B). In contrast, ΔDAD-Daam1 enhanced the formation/maintenance of actin stress fibers in MCF-7 cells (Fig. 6C and 6D). Thus, the findings from the cell biological assay are consistent with the biochemical evidence that RhoA activation required Daam1 activity.

## Discussion

Wnt5a is one of the most highly investigated non-canonical Wnts and has been implicated in almost all aspects of non-canonical Wnt signaling. Wnt5a was able to polarise the cellular cytoskeleton of melanoma cells through a process dependent on dishevelled, RhoB and Rab4 to promote cellular migration towards the source of the CXCL12 chemokine [36]. The second main Wnt5a-dependent pathway is the calcium-dependent signaling pathway, which could have an oncogenic effect by stimulating cancer cell invasion [37]. Wnt5a can also bind and activate the ROR2 tyrosine kinase receptor resulting in the activation of the actin-binding protein and the JNK signaling pathway [38], [39]. In addition to activating non-canonical signaling, Wnt5a is also able to inhibit the activation of the canonical signaling pathway either by calcium signaling through CamK II [40] or through the ROR2 signaling pathways [41]. Wnt5a has ample opportunity to influence cancer cell signaling, resulting in functional promiscuity on cancer development. Wnt5a actually promotes cancer progression and metastasis, such as malignant melanoma and gastric cancer [9], [10], [11]. Previous finding suggested that Wnt5a released from tumor-associated macrophages could have a chemotactic effect on breast cancer cells and thereby increase metastasis [20]. However, some evidence supports the hypothesis that Wnt5a acts as a tumor and developmental suppressor in other breast experimental systems [18], [19], [42], [43].

A primary observation in the present study is that Wnt5a induces the migration of MDA-MB-231 breast cancer cells, which lacks endogenous Wnt5a [22]. It would also be of interest to compare the non-canonical Wnt signaling pathway in normal and breast cancer cells. We found that, similar to the findings by other investigators [22], [44], Wnt5a expression pattern in normal breast cells MCF-10A was similar to that in breast cancer cells (data not shown). However, Wnt5a expression in MCF-10A cells changes in response to *in vitro* growth such as in the presence of EGF, indicating that Wnt5a signaling cannot be reliably studied in such a model system. SFRP2 has been shown to also enhance canonical Wnt signalling, rather than just inhibit it [45], [46], [47], [48]. The transcription of Wnt/β-catenin target genes was increased by forming a complex with LEF/TCF (lymphoid enhancer factor/T cell factor) DNA-binding proteins. We detected the nuclear translocation of β-catenin to illustrate the interaction of sFRP2 and canonical Wnt signaling. SFRP2 does not alter the nuclear translocation of β-catenin in MDA-MB-231 cells.

A large number of studies have indicated that Wnt5a commands a tumor-suppressing effect [13], [19], [49], [50], [51], [52]. A few studies have pointed to Wnt5a having an oncogenic role in tumors arising from a variety of different tissues [11], [53], [54], [55]. Most studies have involved limited sample sets and a significant number have not detailed expression at both the RNA and protein levels. Studies with much larger sample sets will provide the necessary statistical power to validate the extent of the downregulation of Wnt5a in cancer. On the other hand, the potential role for increased Wnt5a expression in malignant melanoma has recently been outlined as a study established that nuclear beta-catenin levels are higher in primary tumors than in metastases and that low expression of nuclear beta-catenin expression in primary tumors predicts poor survival [56]. Metastatic neuroblasts in a xenograft model displayed lower Wnt5a expression than the primary neuroblastoma cells [50]. These suggest that the Wnt5a effect is disparate between primary tumors and metastases. Most studies on Wnt5a expression in cancer patient materials focus on tumorigenesis of primary tumors. Studies of establishing cancer metastases models in cancer patient materials will bring new opinion about the clinical situation.

It has been reported that Wnt5a may promote tumor progression through inducing actin reorganization and increasing cell motility *via* activating the PKC and calcium signaling pathway [10], [36]. However, the roles of Wnt5a/PCP signaling are poorly defined in cancer cells. In this study, we demonstrated that Wnt5a promotes MDA-MB-231 breast cancer cell migration by activating Dvl2/Daam1. Dvl phosphorylation, which is the most proximal signaling event downstream of membrane receptor activation, can be monitored by a shift in the electrophoretic mobility of phosphorylated Dvl [24], [25], [57]. Consistent with these reports, we found that Wnt5a induced Dvl2 phosphorylation rapidly and transiently in MDA-MB-231 cells. Blocking Dvl2 signaling by Dvl2 mutants or siRNA transfection completely abolished Wnt5a-induced cell migration, indicating that Dvl2 activation participates in the regulation of the MDA-MB-231 migration. However, overexpression of Dvl2 did not further promote cell migration, indicating that endogenous Dvl2 is sufficient for mediating Wnt5a-induced cell migration. By immunohistochemistry, Dvl1 was shown to be expressed in 50% of human breast cancers [58]. Wnt5a can also activate Dvl3 by a CK1-dependent mechanism in dopaminergic cells [59]. Whether Dvl1 and Dvl3, homologs of Dvl2, induce the migration of breast cancer cells needs to be further studied.

Daam1 contains multiple regulatory domains. In unstimulated cells, Daam1 exists in an auto-inhibited state by intramolecular interaction between its N-terminal GBD and C-terminal DAD domains [32]. Our results showed that Wnt5a remarkably activated Daam1 in MDA-MB-231 cells. As previously reported, overexpression of *N*-Daam1 inhibits the interaction between Daam1 and Dvl2 [31]. Interference of Daam1 function *via N*-Daam1 overexpression or knockdown of Daam1 expression *via* siRNA transfection inhibited RhoA activation and cell migration by Wnt5a/Dvl2 in MDA-MB-231 cells. But overexpression of *C*-Daam1 and ΔDAD-Daam1, two activated forms of Daam1, did not further promote cell migration. Furthermore, we tested the ability of ΔDAD-Daam1 in stimulating cell migration in a different breast cancer cell line, MCF-7. Constitutively active ΔDAD-Daam1 induced RhoA activation, the formation of stress fibers, and migration of MCF-7 cells, indicating that Daam1 may be specifically required for the activation of Dvl2 after Wnt5a treatment in breast cancer cells.

Wnt signaling activates the small GTPase Rho during *Xenopus* embryogenesis and neurite retraction of mouse neuroblastoma cells [31], [60], [61]. In our study, specific downregulation of Dvl2/Daam1 signaling in MDA-MB-231 cells suppresses Wnt5a-induced activation of RhoA, and upregulation of Daam1 in MCF-7 cells induces RhoA activation. Furthermore, blocking RhoA activity significantly retards Wnt5a-induced stress fiber formation and cell migration. Similarly, previous studies have reported that activated RhoA is critical for breast tumor invasion and metastasis [62], [63], [64]. Therefore, it is possible that Wnt5a-induced RhoA activation may participate in the regulation of MDA-MB-231 and MCF-7 cell migration. Further studies are needed to decipher whether Wnt5a/PCP signaling products function in a common Rho pathway or in parallel pathways.

In summary, we presented the first direct evidence that Wnt5a promotes breast cancer cell migration *via* Dvl2/Daam1/RhoA. These findings elucidate a molecular pathway linking Wnt5a signaling with RhoA in governing cytoskeletal architecture and cell motility, which may represent a rational molecular target for manipulating breast cancer.

## Supporting Information

Figure S1 **Expression of Wnt5a mRNAs and proteins in human breast cancer cell lines.** Total mRNA or protein extracts from MCF-7 and MDA-MB-231 cells were analyzed by RT-PCR (top panel) and immunoblotting (bottom panel) for Wnt5a. The same assay was performed with GAPDH or β-actin as loading control.
(TIF)

Figure S2 **Activity of recombinant Wnt5a (rWnt5a).** rWnt5a was assayed at the indicated doses for electrophoretic mobility shift by immunoblotting using anti-Wnt5a antibodies. The rWnt5a migrates as a single band of an approximately 45 kDa in size.
(TIF)

Figure S3 **Wnt5a promotes MDA-MB-231 cell migration.** MDA-MB-231 cells were stimulated by 500 ng/mL rWnt5a for the indicated time. The cell motility rate was measured by wound healing assay. All values are the mean ± s.d. of 5 independent observations.
(TIF)

Figure S4 **Wnt5a does not appreciably promote MDA-MB-231 cell growth.** Cell proliferation was measured by MTT assays. The mean optical densities of MDA-MB-231 cells are shown. MDA-MB-231 cells were cultured on 96-wells in the absence (Vehicle) or presence of rWnt5a (500 ng/mL). All values are the mean ± s.d. of 5 independent observations.
(TIF)

Figure S5 **sFRP2 does not alter the nuclear translocation of β-catenin in MDA-MB-231 cells.** MDA-MB-231 cells were pre-treated with 1000 ng/mL sFRP2 for 1 h, followed by incubation in the absence or presence of 500 ng/mL rWnt5a for 4 h. Total protein or nucleonic protein extracts from MDA-MB-231 cells were analyzed by immunoblotting for β-catenin. The same assay was performed with histone 3 or β-actin as a loading standard.
(TIF)

Figure S6 **The shifted protein of Dvl2 is the phosphorylated form of Dvl2.** (A) Immunoblot analysis of Dvl2 in MDA-MB-231 cell extracts either untreated (Control), treated with phosphatase, or treated with phosphatase in the presence of phosphatase inhibitor. The mobility shift upon phosphatase treatment confirms that the upper Dvl2 band in MDA-MB-231 cells is hyperphosphorylated. (B) Proteins of MDA-MB-231 cells were immunoprecipitated by anti-Dvl2 antibody, and then were analyzed by blotting with anti-phosphotyrosine, phosphoserine and phosphothreonine antibodies.
(TIF)

Figure S7 **C3 exoenzyme transferase is a specific Rho inhibitor.** MDA-MB-231 cells were pre-treated with 10 ng/µL C3 exoenzyme transferase for 1 h, afterwards incubated in the absence or presence of 500 ng/mL rWnt5a for 30 min. Cells were lysed and quantitated for protein and equal amounts of lysates were assayed for active RhoA by pulldown assays.
(TIF)
