# Supplementary figures and images for: Dvl2-Dependent Activation of Daam1 and RhoA Regulates Wnt5a-Induced Breast Cancer Cell Migration
Source: PLoS One. 2012 May 24;7(5):e37823. doi: 10.1371/journal.pone.0037823 (PMC3360006; doi:10.1371/journal.pone.0037823)

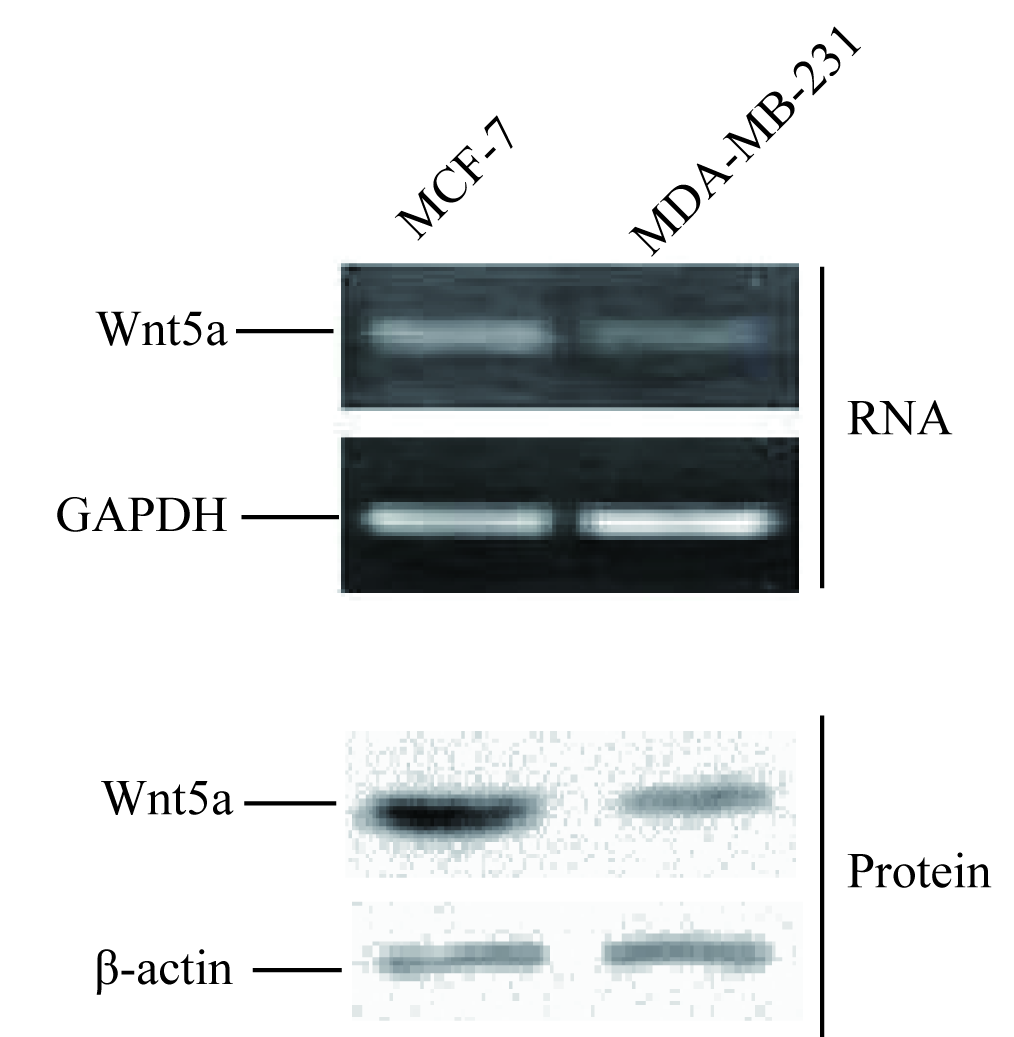

Supplement: Figure S1 — Expression of Wnt5a mRNAs and proteins in human breast cancer cell lines. Total mRNA or protein extracts from MCF-7 and MDA-MB-231 cells were analyzed by RT-PCR (top panel) and immunoblotting (bottom panel) for Wnt5a. The same assay was performed with GAPDH or β-actin as loading control. (TIF) [file pone.0037823.s001.tif]

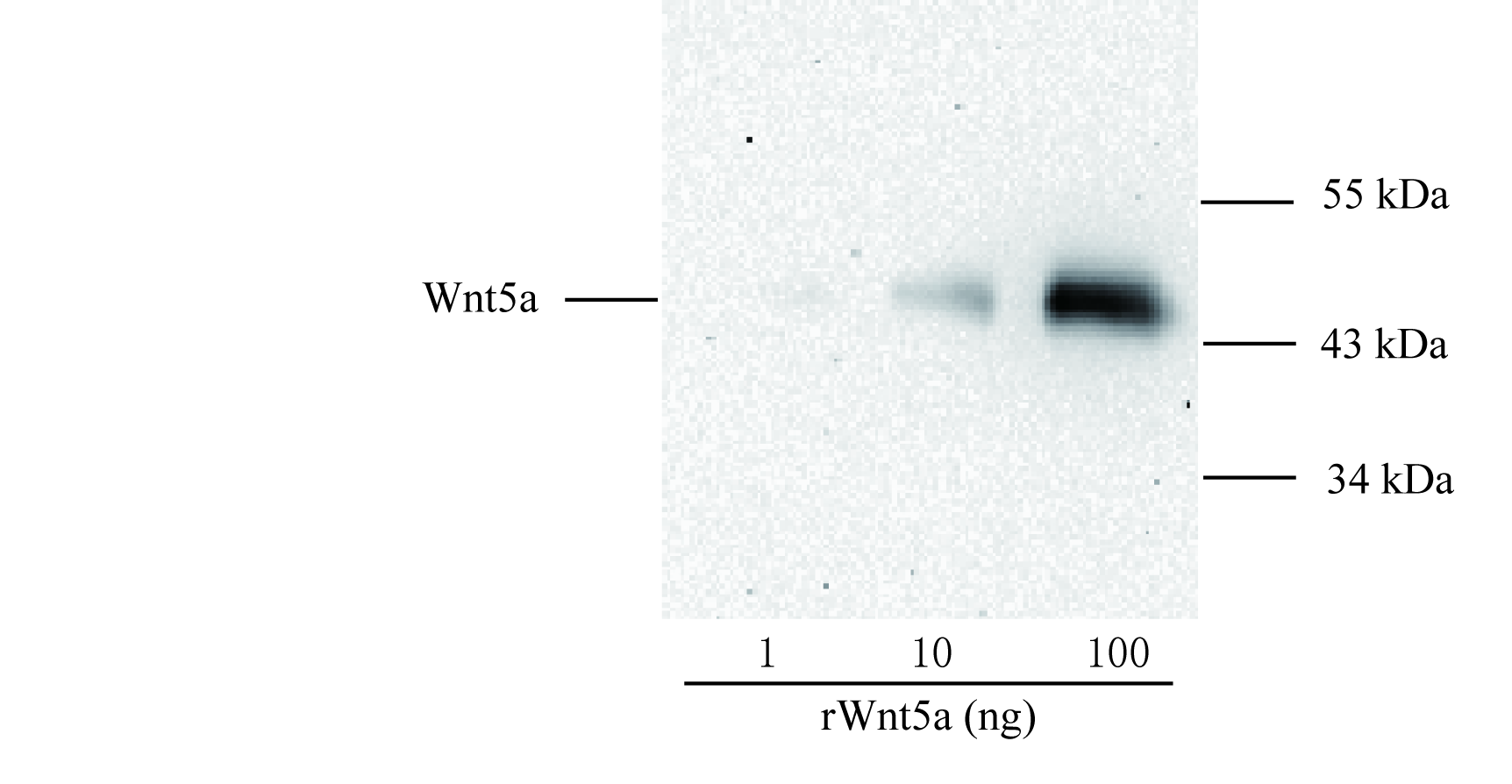

Supplement: Figure S2 — Activity of recombinant Wnt5a (rWnt5a). rWnt5a was assayed at the indicated doses for electrophoretic mobility shift by immunoblotting using anti-Wnt5a antibodies. The rWnt5a migrates as a single band of an approximately 45 kDa in size. (TIF) [file pone.0037823.s002.tif]

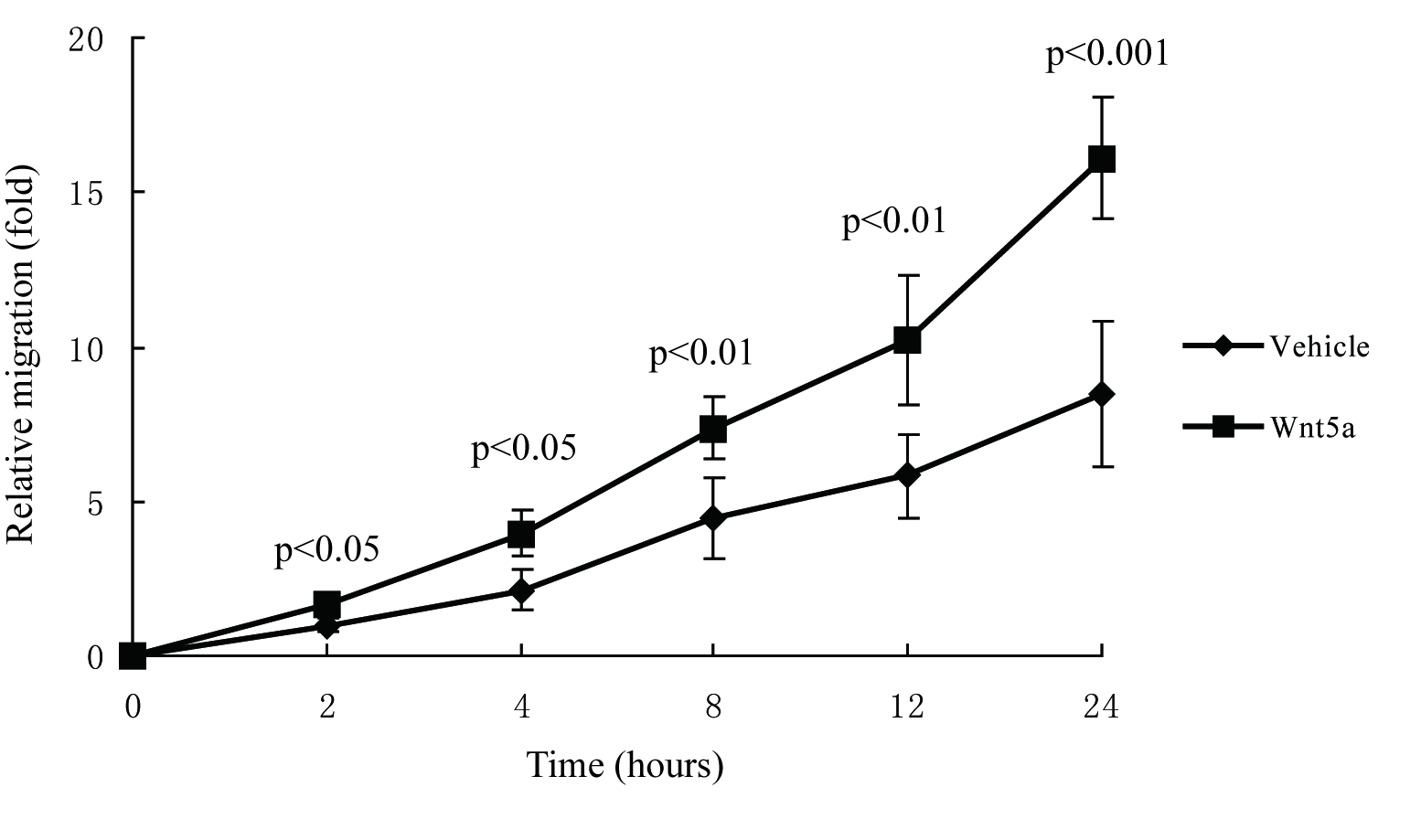

Supplement: Figure S3 — Wnt5a promotes MDA-MB-231 cell migration. MDA-MB-231 cells were stimulated by 500 ng/mL rWnt5a for the indicated time. The cell motility rate was measured by wound healing assay. All values are the mean ± s.d. of 5 independent observations. (TIF) [file pone.0037823.s003.tif]

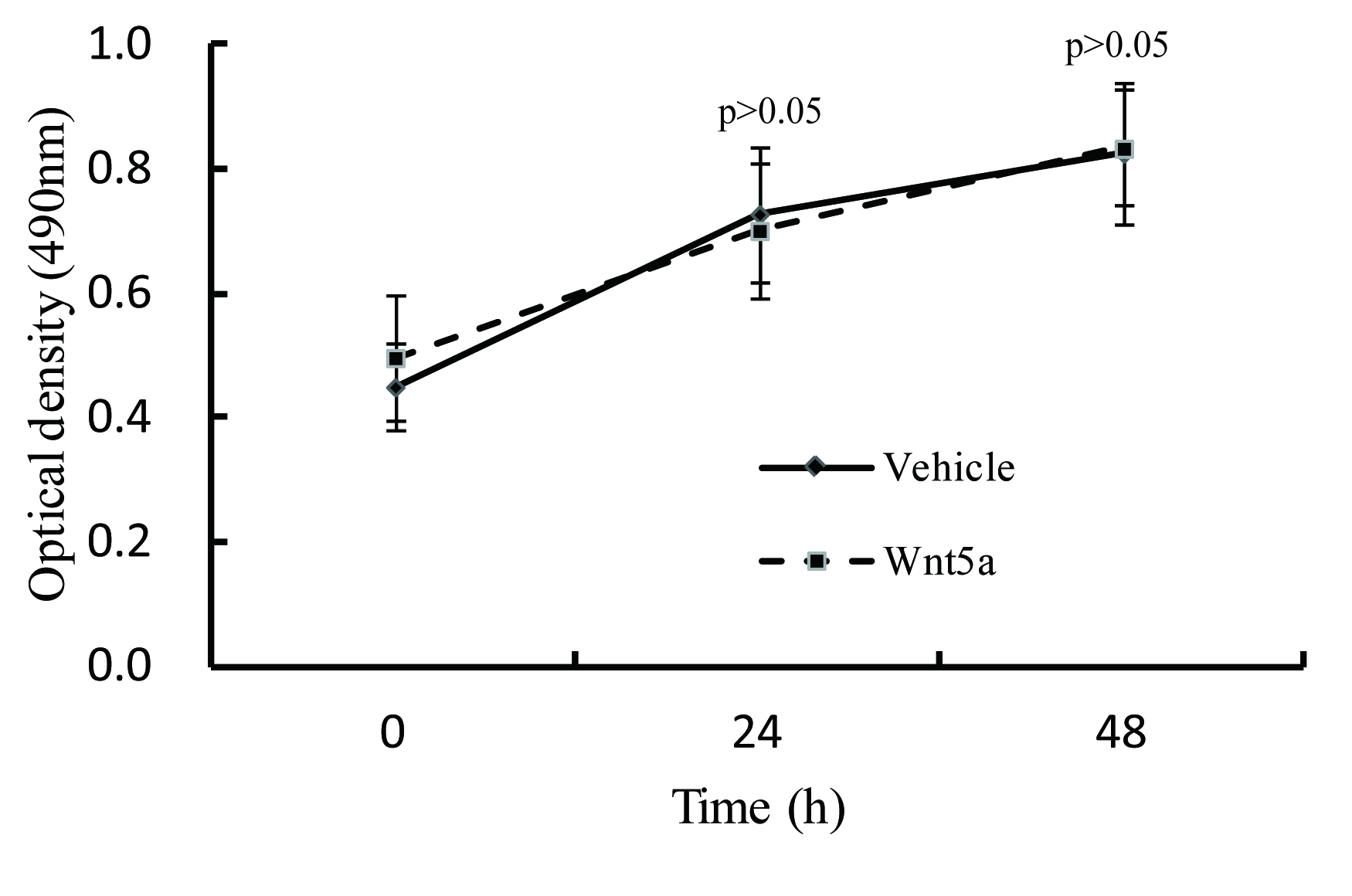

Supplement: Figure S4 — Wnt5a does not appreciably promote MDA-MB-231 cell growth. Cell proliferation was measured by MTT assays. The mean optical densities of MDA-MB-231 cells are shown. MDA-MB-231 cells were cultured on 96-wells in the absence (Vehicle) or presence of rWnt5a (500 ng/mL). All values are the mean ± s.d. of 5 independent observations. (TIF) [file pone.0037823.s004.tif]

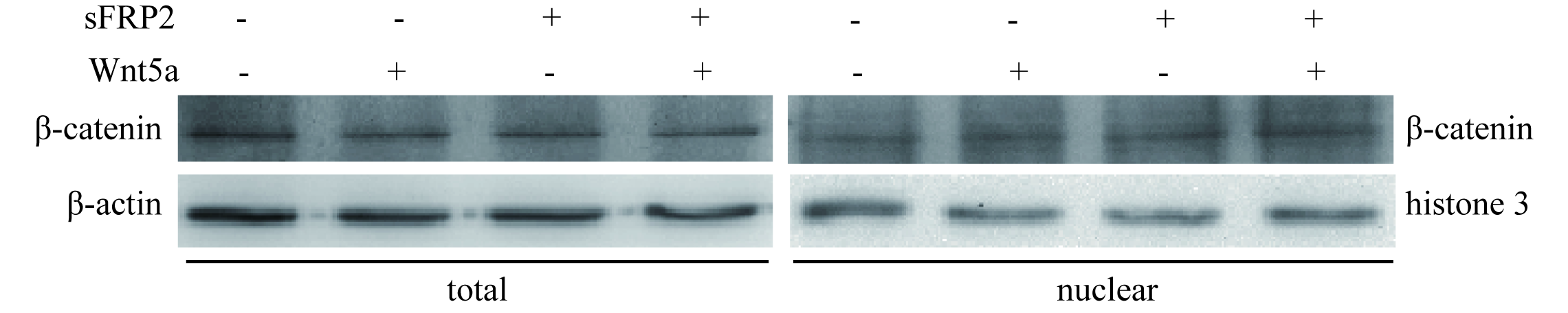

Supplement: Figure S5 — sFRP2 does not alter the nuclear translocation of β-catenin in MDA-MB-231 cells. MDA-MB-231 cells were pre-treated with 1000 ng/mL sFRP2 for 1 h, followed by incubation in the absence or presence of 500 ng/mL rWnt5a for 4 h. Total protein or nucleonic protein extracts from MDA-MB-231 cells were analyzed by immunoblotting for β-catenin. The same assay was performed with histone 3 or β-actin as a loading standard. (TIF) [file pone.0037823.s005.tif]

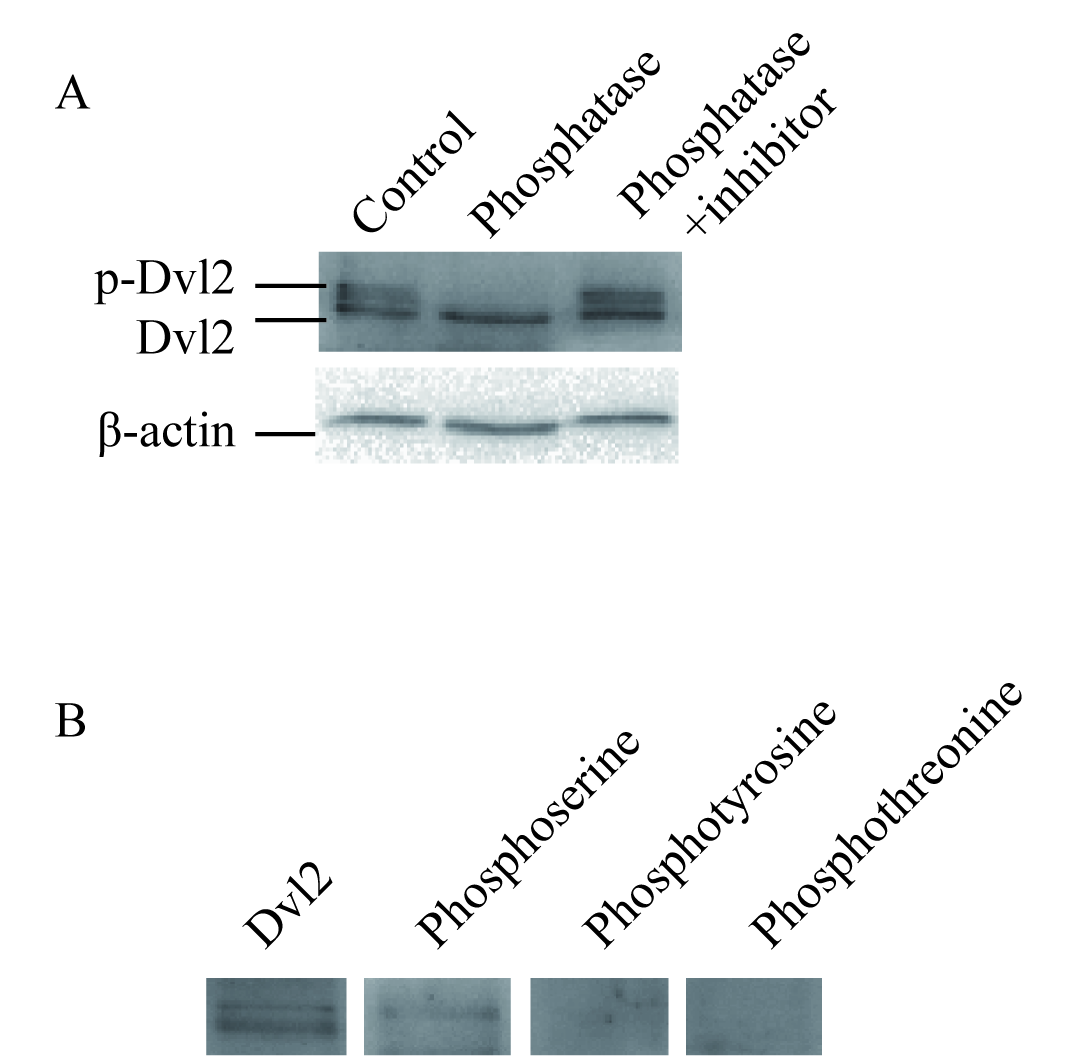

Supplement: Figure S6 — The shifted protein of Dvl2 is the phosphorylated form of Dvl2. (A) Immunoblot analysis of Dvl2 in MDA-MB-231 cell extracts either untreated (Control), treated with phosphatase, or treated with phosphatase in the presence of phosphatase inhibitor. The mobility shift upon phosphatase treatment confirms that the upper Dvl2 band in MDA-MB-231 cells is hyperphosphorylated. (B) Proteins of MDA-MB-231 cells were immunoprecipitated by anti-Dvl2 antibody, and then were analyzed by blotting with anti-phosphotyrosine, phosphoserine and phosphothreonine antibodies. (TIF) [file pone.0037823.s006.tif]

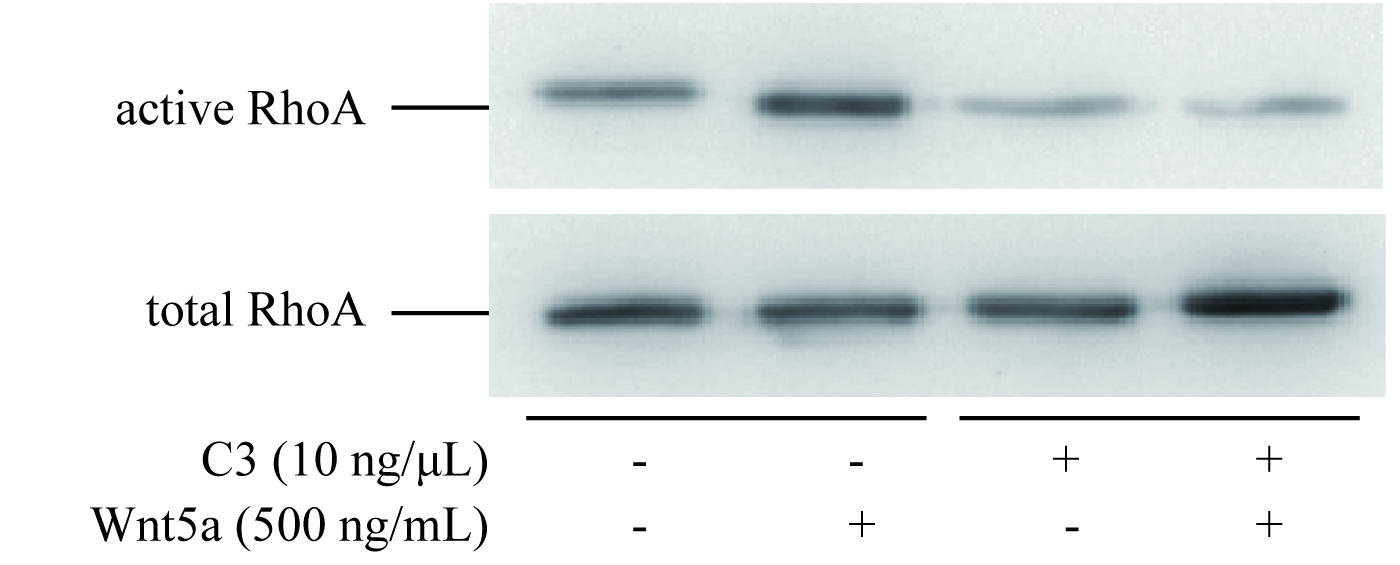

Supplement: Figure S7 — C3 exoenzyme transferase is a specific Rho inhibitor. MDA-MB-231 cells were pre-treated with 10 ng/µL C3 exoenzyme transferase for 1 h, afterwards incubated in the absence or presence of 500 ng/mL rWnt5a for 30 min. Cells were lysed and quantitated for protein and equal amounts of lysates were assayed for active RhoA by pulldown assays. (TIF) [file pone.0037823.s007.tif]
